# 2-Methyl-3-phenyl­sulfinyl-1-benzofuran

**DOI:** 10.1107/S1600536808021107

**Published:** 2008-07-12

**Authors:** Hong Dae Choi, Pil Ja Seo, Byeng Wha Son, Uk Lee

**Affiliations:** a Department of Chemistry, Dongeui University, San 24 Kaya-dong, Busanjin-gu, Busan 614-714, Republic of Korea; b Department of Chemistry, Pukyong National University, 599-1 Daeyeon 3-dong, Nam-gu, Busan 608-737, Republic of Korea

## Abstract

The title compound, C_15_H_12_O_2_S, was prepared by the oxidation of 2-methyl-3-phenyl­sulfanyl-1-benzofuran with 3-chloro­peroxy­benzoic acid. The O atom and the phenyl group of the phenyl­sulfinyl substituent lie on opposite sides of the plane of the benzofuran system. The phenyl ring makes a dihedral angle of 78.76 (4)° with the benzofuran mean plane. The crystal structure is stabilized by π–π inter­actions between the furan and benzene rings of neighbouring mol­ecules [centroid–centroid distance = 4.017 (3) Å]. In addition, the crystal structure exhibits inter­molecular C—H⋯π and C—H⋯O inter­actions.

## Related literature

For the crystal structures of similar substituted benzofuran compounds, see: Choi *et al.* (2007 ▶, 2008 ▶). 
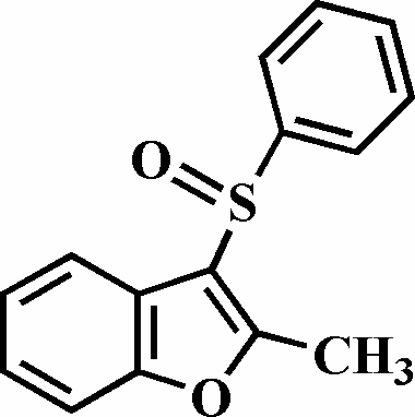


## Experimental

### 

#### Crystal data

C_15_H_12_O_2_S
*M* *_r_* = 256.31
Monoclinic, 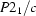

*a* = 12.946 (2) Å
*b* = 9.466 (1) Å
*c* = 10.294 (1) Å
β = 103.887 (2)°
*V* = 1224.6 (3) Å^3^
*Z* = 4
Mo *K*α radiation
μ = 0.25 mm^−1^
*T* = 173 (2) K
0.60 × 0.40 × 0.30 mm

#### Data collection

Bruker SMART CCD diffractometer
Absorption correction: none
6763 measured reflections
2405 independent reflections
2192 reflections with *I* > 2σ(*I*)
*R*_int_ = 0.038

#### Refinement

*R*[*F* ^2^ > 2σ(*F* ^2^)] = 0.038
*wR*(*F* ^2^) = 0.102
*S* = 1.11
2405 reflections
164 parameters
H-atom parameters constrained
Δρ_max_ = 0.25 e Å^−3^
Δρ_min_ = −0.52 e Å^−3^

### 

Data collection: *SMART* (Bruker, 2001 ▶); cell refinement: *SAINT* (Bruker, 2001 ▶); data reduction: *SAINT*; program(s) used to solve structure: *SHELXS97* (Sheldrick, 2008 ▶); program(s) used to refine structure: *SHELXL97* (Sheldrick, 2008 ▶); molecular graphics: *ORTEP-3* (Farrugia, 1997 ▶) and *DIAMOND* (Brandenburg, 1998 ▶); software used to prepare material for publication: *SHELXL97*.

## Supplementary Material

Crystal structure: contains datablocks global, I. DOI: 10.1107/S1600536808021107/gk2156sup1.cif

Structure factors: contains datablocks I. DOI: 10.1107/S1600536808021107/gk2156Isup2.hkl

Additional supplementary materials:  crystallographic information; 3D view; checkCIF report

## Figures and Tables

**Table 1:** Hydrogen-bond geometry (Å, °)

*D*—H⋯*A*	*D*—H	H⋯*A*	*D*⋯*A*	*D*—H⋯*A*
C4—H4⋯O2^i^	0.95	2.59	3.387 (2)	142
C15—H15*C*⋯O2^ii^	0.98	2.42	3.232 (2)	141
C12—H12⋯*Cg*2^iii^	0.95	3.20	3.629 (3)	152

Symmetry codes: (i) 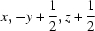; (ii) 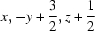; (iii) 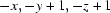. *Cg*2 is the centroid of the C2–C7 benzene ring.

**Fig. 1.**
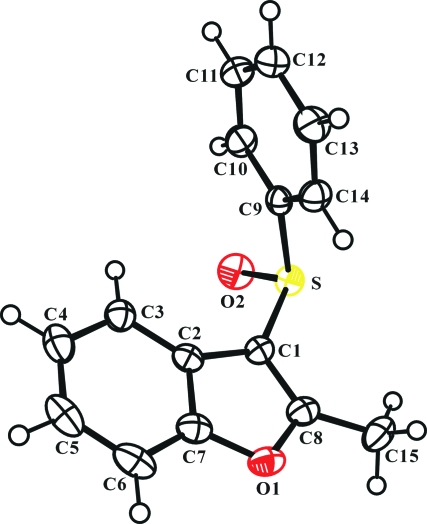
The molecular structure of the title compound, showing displacement ellipsoids drawn at the 50% probability level.

**Fig. 2.**
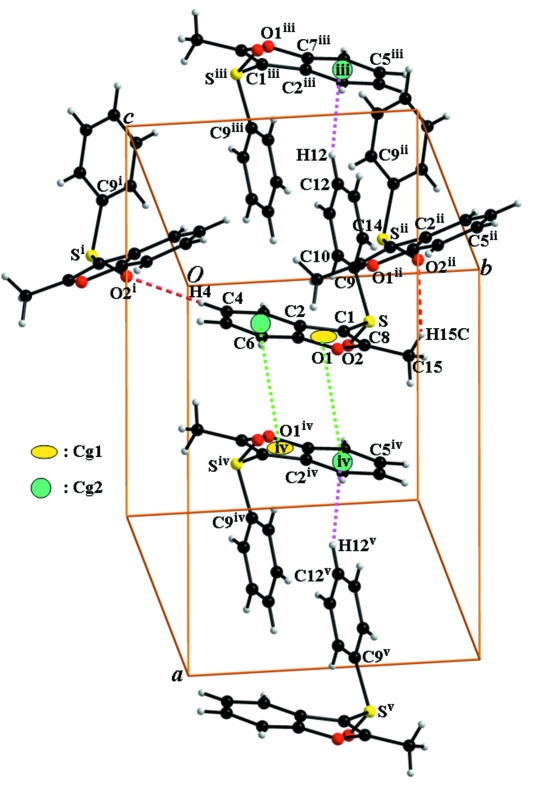
π–π, C—H···π and C—H···O interactions (dotted lines) in the title compound. Cg denotes the ring centroid. [Symmetry codes: (i) x, -y + 1/2, z + 1/2; (ii) x, -y + 3/2, z + 1/2; (iii) -x, -y + 1, -z + 1; (iv) -x + 1, -y + 1, -z + 1; (v) x + 1, y, z.]

## supplementary crystallographic information

### Comment

This work is related to our communications on the synthesis and structures of 3-phenylsulfinyl-1-benzofuran analogues, *viz*. 2,5-dimethyl-3-phenylsulfinyl-1-benzofuran (Choi *et al.*, 2007) and 2,4,6,7-tetramethyl-3-phenylsulfinyl-1-benzofuran (Choi *et al.*, 2008). Here we report the crystal structure of the title compound, 2-methyl-3-phenylsulfinyl-1-benzofuran (Fig. 1).

The benzofuran unit is almost planar, with a mean deviation of 0.008 (1) ° from the least-squares plane defined by the nine constituent atoms. The phenyl ring (C9—C14) is almost perpendicular to the plane of the benzofuran ring system [78.76 (4)°] and is tilted slightly towards it. The crystal packing (Fig. 2) is stabilized by aromatic π–π interactions between the furan and the benzene rings of neighbouring molecules. The *Cg*1···*Cg*2^iv^ distance is 4.017 (3) Å (*Cg*1 and *Cg*2 are the centroids of the C1/C2/C7/O1/C8 furan ring and the C2—C7 benzene ring, respectively, symmetry code as in Fig. 2). The crystal packing is further stabilized by C—H···π interactions between a phenyl H atom of the phenylsulfinyl substitutent and the benzene ring of the benzofuran unit, with a C12-H12···Cg2^iii^ separation of 3.20 Å (Fig. 2 and Table 1; Cg2 is the centroid of the C2-C7 benzene ring, symmetry code as in Fig. 2). Additionally, intermolecular C—H···O interactions in the structure were observed (Fig. 2 and Table 1; symmetry code as in Fig. 2).

### Experimental

77% 3-Chloroperoxybenzoic acid (247 mg, 1.1 mmol) was added in small portions to a stirred solution of 2-methyl-3-phenylsulfanyl-1-benzofuran (240 mg, 1.0 mmol) in dichloromethane (30 ml) at 273 K. After being stirred for 3 h at room temperature, the mixture was washed with saturated sodium bicarbonate solution and the organic layer was separated, dried over magnesium sulfate, filtered and concentrated under vacuum. The residue was purified by column chromatography (hexane-ethyl acetate, 1:2 *v*/*v*) to afford the title compound as a colorless solid [yield 78%, m.p. 417–418 K; *R*_f_ = 0.71 (hexane-ethyl acetate, 1:2 *v*/*v*)]. Single crystals suitable for X-ray diffraction were prepared by evaporation of a solution of the title compound in acetone at room temperature. Spectroscopic analysis: ^1^H NMR (CDCl_3_, 400 MHz) δ 2.77 (s, 3H), 7.06 (t, J = 7.80 Hz, 1H), 7.19–7.23 (m, 2H), 7.39–7.52 (m, 4H), 7.68 (d, J = 7.44 Hz, 2H); EI—MS 256 [*M*^+^].

### Refinement

All H atoms were positioned geometrically and refined using a riding model, with C—H = 0.95 Å for aromatic H atoms and 0.98 Å for methyl H atoms, respectively, and with *U*_iso_(H) = 1.2Ueq(C) for aromatic and *U*_iso_(H) = 1.5Ueq(C) for methyl H atoms.

### Crystal data

**Table:**

C_15_H_12_O_2_S	*F*_000_ = 536
*M**_r_* = 256.31	*D*_x_ = 1.390 Mg m^−^^3^
Monoclinic, *P*2_1_/*c*	Mo *K*α radiation λ = 0.71073 Å
Hall symbol: -P_2ybc	Cell parameters from 5415 reflections
*a* = 12.946 (2) Å	θ = 2.7–28.2º
*b* = 9.466 (1) Å	µ = 0.25 mm^−^^1^
*c* = 10.294 (1) Å	*T* = 173 (2) K
β = 103.887 (2)º	Block, colorless
*V* = 1224.6 (3) Å^3^	0.60 × 0.40 × 0.30 mm
*Z* = 4	

### Data collection

**Table:**

Bruker SMART CCD diffractometer	2405 independent reflections
Radiation source: fine-focus sealed tube	2192 reflections with *I* > 2σ(*I*)
Monochromator: graphite	*R*_int_ = 0.038
Detector resolution: 10.0 pixels mm^-1^	θ_max_ = 26.0º
*T* = 173(2) K	θ_min_ = 1.6º
φ and ω scans	*h* = −15→15
Absorption correction: none	*k* = −11→7
6763 measured reflections	*l* = −12→12

### Refinement

**Table:**

Refinement on *F*^2^	Secondary atom site location: difference Fourier map
Least-squares matrix: full	Hydrogen site location: inferred from neighbouring sites
*R*[*F*^2^ > 2σ(*F*^2^)] = 0.039	H-atom parameters constrained
*wR*(*F*^2^) = 0.102	*w* = 1/[σ^2^(*F*_o_^2^) + (0.0544*P*)^2^ + 0.4838*P*] where *P* = (*F*_o_^2^ + 2*F*_c_^2^)/3
*S* = 1.11	(Δ/σ)_max_ = 0.001
2405 reflections	Δρ_max_ = 0.25 e Å^−^^3^
164 parameters	Δρ_min_ = −0.52 e Å^−^^3^
Primary atom site location: structure-invariant direct methods	Extinction correction: none

### Special details

**Table:**

Geometry. All e.s.d.'s (except the e.s.d. in the dihedral angle between two l.s. planes) are estimated using the full covariance matrix. The cell e.s.d.'s are taken into account individually in the estimation of e.s.d.'s in distances, angles and torsion angles; correlations between e.s.d.'s in cell parameters are only used when they are defined by crystal symmetry. An approximate (isotropic) treatment of cell e.s.d.'s is used for estimating e.s.d.'s involving l.s. planes.
Refinement. Refinement of *F*^2^ against ALL reflections. The weighted *R*-factor *wR* and goodness of fit *S* are based on *F*^2^, conventional *R*-factors *R* are based on *F*, with *F* set to zero for negative *F*^2^. The threshold expression of *F*^2^ > σ(*F*^2^) is used only for calculating *R*-factors(gt) *etc*. and is not relevant to the choice of reflections for refinement. *R*-factors based on *F*^2^ are statistically about twice as large as those based on *F*, and *R*- factors based on ALL data will be even larger.

### Fractional atomic coordinates and isotropic or equivalent isotropic displacement parameters (Å^2^)

**Table:**

	*x*	*y*	*z*	*U*_iso_*/*U*_eq_	
S	0.21943 (3)	0.67373 (4)	0.24290 (4)	0.02584 (14)	
O1	0.42892 (9)	0.63644 (14)	0.58590 (11)	0.0324 (3)	
O2	0.23114 (10)	0.57743 (14)	0.13205 (11)	0.0372 (3)	
C1	0.30363 (12)	0.61722 (17)	0.39339 (15)	0.0239 (3)	
C2	0.31131 (11)	0.48127 (17)	0.45930 (14)	0.0244 (3)	
C3	0.26168 (13)	0.35014 (17)	0.43207 (16)	0.0287 (3)	
H3	0.2089	0.3341	0.3517	0.034*	
C4	0.29212 (14)	0.24396 (19)	0.52657 (18)	0.0353 (4)	
H4	0.2590	0.1540	0.5107	0.042*	
C5	0.37026 (15)	0.2664 (2)	0.64428 (18)	0.0394 (4)	
H5	0.3885	0.1916	0.7071	0.047*	
C6	0.42179 (14)	0.3951 (2)	0.67166 (17)	0.0379 (4)	
H6	0.4760	0.4104	0.7507	0.045*	
C7	0.38969 (12)	0.49963 (19)	0.57741 (15)	0.0291 (4)	
C8	0.37502 (12)	0.70491 (18)	0.47218 (15)	0.0277 (3)	
C9	0.09419 (12)	0.62970 (16)	0.27953 (14)	0.0235 (3)	
C10	0.02207 (13)	0.55290 (18)	0.18570 (16)	0.0317 (4)	
H10	0.0412	0.5168	0.1086	0.038*	
C11	−0.07920 (14)	0.5289 (2)	0.20534 (19)	0.0374 (4)	
H11	−0.1296	0.4765	0.1412	0.045*	
C12	−0.10656 (13)	0.58118 (19)	0.31772 (19)	0.0362 (4)	
H12	−0.1759	0.5651	0.3306	0.043*	
C13	−0.03302 (14)	0.65707 (19)	0.41178 (18)	0.0359 (4)	
H13	−0.0519	0.6921	0.4894	0.043*	
C14	0.06790 (13)	0.68229 (17)	0.39348 (16)	0.0295 (4)	
H14	0.1183	0.7347	0.4578	0.035*	
C15	0.40351 (14)	0.85446 (19)	0.45966 (19)	0.0364 (4)	
H15A	0.3707	0.8880	0.3691	0.055*	
H15B	0.4810	0.8633	0.4766	0.055*	
H15C	0.3778	0.9113	0.5250	0.055*	

### Atomic displacement parameters (Å^2^)

**Table:**

	*U*^11^	*U*^22^	*U*^33^	*U*^12^	*U*^13^	*U*^23^
S	0.0309 (2)	0.0267 (2)	0.0210 (2)	−0.00105 (15)	0.00834 (16)	0.00241 (14)
O1	0.0259 (6)	0.0415 (7)	0.0281 (6)	−0.0028 (5)	0.0033 (5)	−0.0067 (5)
O2	0.0444 (7)	0.0478 (8)	0.0227 (6)	0.0018 (6)	0.0143 (5)	−0.0049 (5)
C1	0.0239 (7)	0.0266 (8)	0.0227 (7)	−0.0017 (6)	0.0083 (6)	−0.0008 (6)
C2	0.0223 (7)	0.0298 (8)	0.0225 (7)	0.0032 (6)	0.0082 (6)	0.0006 (6)
C3	0.0291 (8)	0.0281 (8)	0.0300 (8)	0.0009 (6)	0.0094 (6)	−0.0002 (6)
C4	0.0382 (9)	0.0290 (9)	0.0431 (9)	0.0080 (7)	0.0184 (8)	0.0058 (7)
C5	0.0419 (10)	0.0439 (11)	0.0363 (9)	0.0196 (8)	0.0171 (8)	0.0148 (8)
C6	0.0333 (9)	0.0535 (11)	0.0260 (8)	0.0157 (8)	0.0056 (7)	0.0036 (8)
C7	0.0244 (7)	0.0381 (9)	0.0260 (8)	0.0029 (7)	0.0082 (6)	−0.0034 (7)
C8	0.0234 (7)	0.0340 (9)	0.0278 (8)	−0.0023 (6)	0.0103 (6)	−0.0051 (7)
C9	0.0270 (8)	0.0215 (7)	0.0211 (7)	0.0020 (6)	0.0041 (6)	0.0024 (6)
C10	0.0346 (8)	0.0323 (9)	0.0257 (8)	0.0012 (7)	0.0026 (6)	−0.0051 (7)
C11	0.0301 (9)	0.0344 (9)	0.0422 (9)	−0.0027 (7)	−0.0023 (7)	−0.0063 (8)
C12	0.0264 (8)	0.0342 (9)	0.0480 (10)	0.0003 (7)	0.0086 (7)	0.0015 (8)
C13	0.0353 (9)	0.0392 (10)	0.0358 (9)	0.0014 (7)	0.0141 (7)	−0.0051 (7)
C14	0.0303 (8)	0.0317 (9)	0.0263 (8)	−0.0020 (6)	0.0062 (6)	−0.0061 (6)
C15	0.0356 (9)	0.0336 (9)	0.0441 (10)	−0.0114 (7)	0.0175 (8)	−0.0116 (8)

### Geometric parameters (Å, °)

**Table:**

S—O2	1.496 (1)	C6—H6	0.9500
S—C1	1.750 (2)	C8—C15	1.476 (2)
S—C9	1.800 (2)	C9—C10	1.378 (2)
O1—C8	1.373 (2)	C9—C14	1.390 (2)
O1—C7	1.386 (2)	C10—C11	1.392 (2)
C1—C8	1.357 (2)	C10—H10	0.9500
C1—C2	1.447 (2)	C11—C12	1.380 (3)
C2—C3	1.395 (2)	C11—H11	0.9500
C2—C7	1.395 (2)	C12—C13	1.385 (3)
C3—C4	1.388 (2)	C12—H12	0.9500
C3—H3	0.9500	C13—C14	1.385 (2)
C4—C5	1.396 (3)	C13—H13	0.9500
C4—H4	0.9500	C14—H14	0.9500
C5—C6	1.385 (3)	C15—H15A	0.9800
C5—H5	0.9500	C15—H15B	0.9800
C6—C7	1.378 (2)	C15—H15C	0.9800
			
O2—S—C1	109.46 (7)	C1—C8—C15	133.1 (2)
O2—S—C9	106.26 (7)	O1—C8—C15	116.1 (1)
C1—S—C9	98.33 (7)	C10—C9—C14	121.2 (2)
C8—O1—C7	106.5 (1)	C10—C9—S	117.9 (1)
C8—C1—C2	107.7 (1)	C14—C9—S	120.6 (1)
C8—C1—S	122.2 (1)	C9—C10—C11	119.2 (2)
C2—C1—S	130.2 (1)	C9—C10—H10	120.4
C3—C2—C7	119.2 (2)	C11—C10—H10	120.4
C3—C2—C1	136.1 (1)	C12—C11—C10	120.2 (2)
C7—C2—C1	104.6 (1)	C12—C11—H11	119.9
C4—C3—C2	117.6 (2)	C10—C11—H11	119.9
C4—C3—H3	121.2	C11—C12—C13	120.0 (2)
C2—C3—H3	121.2	C11—C12—H12	120.0
C3—C4—C5	121.5 (2)	C13—C12—H12	120.0
C3—C4—H4	119.2	C14—C13—C12	120.5 (2)
C5—C4—H4	119.2	C14—C13—H13	119.8
C6—C5—C4	121.6 (2)	C12—C13—H13	119.8
C6—C5—H5	119.2	C13—C14—C9	118.9 (2)
C4—C5—H5	119.2	C13—C14—H14	120.6
C7—C6—C5	116.1 (2)	C9—C14—H14	120.6
C7—C6—H6	122.0	C8—C15—H15A	109.5
C5—C6—H6	122.0	C8—C15—H15B	109.5
C6—C7—O1	125.6 (2)	H15A—C15—H15B	109.5
C6—C7—C2	123.9 (2)	C8—C15—H15C	109.5
O1—C7—C2	110.5 (1)	H15A—C15—H15C	109.5
C1—C8—O1	110.8 (2)	H15B—C15—H15C	109.5
			
O2—S—C1—C8	126.3 (1)	C1—C2—C7—O1	−0.1 (2)
C9—S—C1—C8	−123.2 (1)	C2—C1—C8—O1	−0.3 (2)
O2—S—C1—C2	−55.6 (2)	S—C1—C8—O1	178.2 (1)
C9—S—C1—C2	55.0 (2)	C2—C1—C8—C15	−179.5 (2)
C8—C1—C2—C3	−179.9 (2)	S—C1—C8—C15	−1.0 (3)
S—C1—C2—C3	1.7 (3)	C7—O1—C8—C1	0.3 (2)
C8—C1—C2—C7	0.2 (2)	C7—O1—C8—C15	179.6 (1)
S—C1—C2—C7	−178.1 (1)	O2—S—C9—C10	−17.0 (2)
C7—C2—C3—C4	1.1 (2)	C1—S—C9—C10	−130.2 (1)
C1—C2—C3—C4	−178.7 (2)	O2—S—C9—C14	168.5 (1)
C2—C3—C4—C5	−0.6 (2)	C1—S—C9—C14	55.3 (1)
C3—C4—C5—C6	−0.7 (3)	C14—C9—C10—C11	0.6 (2)
C4—C5—C6—C7	1.3 (2)	S—C9—C10—C11	−173.9 (1)
C5—C6—C7—O1	178.7 (1)	C9—C10—C11—C12	−0.2 (3)
C5—C6—C7—C2	−0.8 (2)	C10—C11—C12—C13	−0.3 (3)
C8—O1—C7—C6	−179.6 (2)	C11—C12—C13—C14	0.6 (3)
C8—O1—C7—C2	−0.1 (2)	C12—C13—C14—C9	−0.3 (3)
C3—C2—C7—C6	−0.4 (2)	C10—C9—C14—C13	−0.3 (2)
C1—C2—C7—C6	179.4 (2)	S—C9—C14—C13	174.0 (1)
C3—C2—C7—O1	−179.9 (1)		

### Hydrogen-bond geometry (Å, °)

**Table:**

*D*—H···*A*	*D*—H	H···*A*	*D*···*A*	*D*—H···*A*
C4—H4···O2^i^	0.95	2.59	3.387 (2)	142
C15—H15C···O2^ii^	0.98	2.42	3.232 (2)	141
C12—H12···Cg2^iii^	0.95	3.20	3.629 (3)	152

Symmetry codes: (i) *x*, −*y*+1/2, *z*+1/2; (ii) *x*, −*y*+3/2, *z*+1/2; (iii) −*x*, −*y*+1, −*z*+1.
